# Understanding the role of sex on outcomes for the cancer patient undergoing treatment with immune checkpoint inhibitors: a scoping review protocol

**DOI:** 10.1136/bmjopen-2021-059782

**Published:** 2022-07-21

**Authors:** Amy L Shaver, Nikita Nikita, Swapnil Sharma, Daniel S Lefler, Atrayee Basu-Mallick, Jennifer M Johnson, Meghan L Butryn, Grace Lu-Yao

**Affiliations:** 1 Department of Medical Oncology, Thomas Jefferson University - Sidney Kimmel Medical College, Philadelphia, Pennsylvania, USA; 2 Department of Psychology, Drexel University, Philadelphia, Pennsylvania, USA; 3 Jefferson College of Population Health, Philadelphia, Pennsylvania, USA

**Keywords:** oncology, immunology, epidemiology

## Abstract

**Introduction:**

Immune checkpoint inhibitors (ICIs) have changed the treatment landscape for multiple cancer types. Sex plays an important role in both the development of cancer as well as the functioning of the immune system. Though a difference in response to immune therapy is emerging between men and women it is unclear how this difference affects cancer outcomes and what the potential underlying mechanisms are for those effects. The objective of this study is to describe the influence that sex has on the outcomes experienced by cancer patients on ICI therapy and to identify and analyse any knowledge gaps in the field.

**Method and analysis:**

The framework for this methodology was guided by the Joanna Briggs Institute Manual for Evidence Synthesis. The search and review will be conducted from January 2022 to June 2022. Two independent researchers will screen titles and abstracts followed by full-text screening for manuscript inclusion. Full length studies published between 2010 and December 2021 found in PubMed, Cochrane, CINAHL, and Scopus describing the influence of sex differences on cancer outcomes in patients treated with ICIs will be included. After data are extracted it will be summarised for presentation.

**Ethics and dissemination:**

The findings of this scoping review will be published in a peer-reviewed journal. The results will be used to inform future studies on the potential differential impacts of ICIs. All data are from published openly accessible sources and therefore no ethical clearance is necessary.

**Strengths and limitations of this study**

- Multidisciplinary team used during the planning and design.
- Multiple databases used to source literature.
- No formal quantitative synthesis completed without sufficient data.

## Introduction

Immune checkpoint inhibitors (ICIs) have demonstrated clinical efficacy in several solid tumour types.^1^ Furthermore, the role of ICIs is going to be investigated in the neoadjuvant, adjuvant and definitive settings in ongoing clinical trials making it not only important to understand how to improve patient’s abilities to respond to treatment as it expands its role in multiple cancer types.

Sex effects both the innate and adaptive immune system responses, however less than 10% of all immunology-related studies consider sex while reporting results.^2 3^ Sex hormones have been shown to influence immune system functioning. Among patients with autoimmune diseases medications used to suppress immune response were more efficacious in men than women while medications to stimulate response are more efficacious in women.^4^ Sex plays an important role in the pathogenesis and prognosis of several cancer types. For a vast majority of cancer types, men have a higher predisposition of developing cancer than women. Specifically, men have a twofold higher risk of mortality from all malignant cancer types than women (after excluding sex-specific cancers such as breast and prostate).^5^ Women are under-represented in randomised clinical trials (RCTs) for immunotherapies. In a meta-analysis of phase II and III immunotherapy, RCTs observed improvement in overall survival and progression free survival for both men and women, but the benefit was much larger among men than in women.^6–8^ ICIs depend on antigen presentation occurring. However, women sometimes have a lower tumour mutation burden.^9^ Another study discovered that in female antigens are not as frequently presented to the immune system by the major histocompatibility complex.^10^ Altogether, this may explain why ICIs become less effectual.^11^ Since evidence suggests that men and women respond differently to therapies, it is important to explore these differences to redefine clinical decision making and improve outcomes for all patients with cancer. A first step of that discovery is through an examination of the current evidence.

The purpose of this scoping review is to describe the influence that sex has on the outcomes experienced by patients with cancer on ICI therapy and to identify and analyse any knowledge gaps in the field.

## Methods

### Protocol design

This scoping review will be conducted according to the Joanna Briggs Institute methodology for scoping reviews which is based predominately on the protocols established by Arksey and O’Malley but includes the revisions suggested by Levac *et al* and Peters *et al*.^12–16^ The review will follow six steps including: (1) defining the research question, (2) identifying relevant studies, (3) study selection, (4) charting the data, (5) collating, summarising, and reporting the results, and (6) consultation. Reporting of findings will be conducted according to Preferred Reporting Items for Systematic Reviews and Meta-Analyses (PRISMA) guidelines using the PRISMA extension for scoping reviews (PRISMA-ScR) checklist.^17^

### Stage 1: identifying the research question

The central research question for this review is: what influence does sex play on the outcomes experienced by patients with cancer on immune checkpoint inhibitor therapy? Outcomes are being left purposefully vague so as to capture as much information as possible. All outcome data will then be categorised later in the ‘collating, summarising and reporting’ stage. Also to be examined is whether the ICI therapy is monotherapy, dual therapy or combination with other therapies.

### Stage 2: identifying relevant studies, search strategy

The search strategy for this review is the result of prior research in the adjacent fields of prostate cancer, lung cancer, and immunotherapy as well as the strategies recommended by Tawfik *et al* for adapting searches according to database.^18^ An experienced search librarian was also consulted. We will conduct a search of PubMed, CINAHL, Cochrane Library and Scopus.

We will conduct a search using the following keywords: cancer, “neoplasms”, “immune checkpoint inhibitors”, “sex”, “sex factors”, and associated mesh terms. These terms will be combined with the Boolean operators “AND” and “OR”.

The initial search will be in PubMed. A similar search will be used for Cochrane which also uses MeSH terms. Only key words will be used for SCOPUS. CINAHL uses Subject terms in lieu of MeSH terms.

The search string for the PubMed database is as follows: cancer* OR (“Neoplasms”[Mesh])

AND

“Sex”[MeSH Terms] OR “Sex Characteristics”[MeSH Terms] OR “Sex Factors”[MeSH Terms] OR “Male”[MeSH Terms] OR “Female”[MeSH Terms] OR “sex-related” OR “gender-related” OR “sex differences”

AND

“Immune checkpoint inhibitor” OR “anti-PD-1” OR “anti-CTLA-4” OR “anti-PD-L1” OR “Immune checkpoint blockade” OR “REGN2810” OR “ Cemiplimab” OR “BMS-936559” OR “MSB0010718C” OR “avelumab” OR “MEDI4736” OR “durvalumab” OR MPDL3280A” OR “atezolizumab” OR “MK-3475” OR “pembrolizumab” OR “BMS-936558” OR “nivolumab”

### Stage 3: study selection

#### Inclusion criteria

##### Types of participants

This scoping review will only include adult patients aged 18 years or olderbeing treated with ICIs for those cancers for which the Food and Drug Administration has approved the use of ICIs as treatment as of December 2020.

##### Concept

The review will focus on peer-reviewed publications that seek to clarify the influence of sex on the outcomes of ICIs.

##### Context

The review will include both institutional and community care settings.

##### Types of sources

All peer-reviewed publications published since 2010 through December 2021.

#### Exclusion criteria

The search will be restricted to articles and reports published in English.

Opinion pieces.

Letters to the editor.

Studies identified by these terms which satisfy the inclusion criteria will be considered for the initial title and abstract screening. The search string will be adapted for other databases as required. The reference lists of all included articles will be searched for additional studies. As required by good practice, the completed strings for each database will be included in the published scoping review.

If further information is required, we will contact authors of the publications as appropriate.

The research team will use Endnote V.X9 software for managing imported references and removing duplications. The title, abstracts and keywords for all articles will be screened by two independent reviewers from the research team to determine whether they satisfy the inclusion criteria, see Inclusion Assessment Form, online supplemental material. Each article will be considered by at least two researchers for inclusion and any discrepancies will be will be discussed.

10.1136/bmjopen-2021-059782.supp1 Supplementary data

Articles satisfying the initial screen will undergo full text screening by two independent researchers from the research team. An important part of full-text screening is to discern the inclusion of sex comparison, which my only occur through covariate inclusion but still offer comparison between the sexes. Disagreements of study eligibility will be resolved through discussion with a senior member of the research team.

### Stage 4: charting the data

#### Extraction of the results

Three members of the research team will participate in the data extraction process. From each article, the following information will be extracted: author, year of publication, title, ICI, study type/design, study population, primary objective(s) and outcome(s)/summary, see Extraction Charting Form, online supplemental material.

10.1136/bmjopen-2021-059782.supp2 Supplementary data

### Patient and public involvement

This research will be done without patient involvement. Patients are neither invited to comment on the study design nor consulted to develop patient-relevant outcomes nor to interpret nor disseminate the results. Sex differences in cancer outcomes are known to be important to patients. Any future studies deriving from this work will include patient involvement to help to ensure that, from a patient’s perspective, the outcomes are relevant.

### Stage 5: collating, summarising and reporting

#### Presentation of our results

Our search results will be presented in a PRISMA flowchart and an appended PRISMA-ScR checklist. The extracted data will be presented under the following headings: author, year of publication, study type, study population, primary objective(s) and outcome(s).

A full summary of evidence including an overview of concepts and types of evidence available as well as a discussion of limitations and our conclusions will follow. Analyses of mono and combination therapies will be conducted separately when possible. We will identify gaps in the literature and highlight the implications for future research.

### Stage 6: consultation

We have purposefully included researchers from multiple disciplines in our group (pharmacy, medicine and epidemiology). The diversity of the group brings fresh views and broad experiences to the analysis of the literature. At the end of the study, a final consultation will take place so the results of the study can have the context of clinical practice knowledge.

### Ethics and dissemination

The scoping review as indicated earlier is based on openly accessible published material and is therefore not subject to an ethical review board. The findings of this scoping review will be published in a peer-reviewed journal. The results will be used to inform future studies on the potential differential impacts of ICIs.

## Supplementary Material

Reviewer comments

Author's manuscript
