# Supplementary material for: Understanding the role of sex on outcomes for the cancer patient undergoing treatment with immune checkpoint inhibitors: a scoping review protocol
Source: BMJ Open. 2022 Jul 21;12(7):e059782. doi: 10.1136/bmjopen-2021-059782 (PMC9310149; doi:10.1136/bmjopen-2021-059782)
Supplement: Supplementary data [file bmjopen-2021-059782supp001.pdf]

Inclusion Assessment Form

Sex and immune checkpoint inhibitor (ICI) treatment

Reviewer: Date:

Reference:

Instructions: Complete the form on each study. If the final decision is "no", exclude that study.

CRITERIA

		Yes	No	Unsure
1. Study Publication				
	Is the study a full manuscript?	[ ]	[ ]	[ ]
	Was the study published between January 2010 and December 2021?	[ ]	[ ]	[ ]
	Was the study published in English?	[ ]	[ ]	[ ]
2. Study Population				
	Did the study include patients aged 18 and over?	[ ]	[ ]	[ ]
3. Study Exposure				
	Were patients receiving treatment with an ICI?	[ ]	[ ]	[ ]
	Does the study include both males and females?	[ ]	[ ]	[ ]
4. Study Outcomes				
	Did the study report on one or more outcomes?<br>i.e. The study is not an opinion, editorial, etc. only	[ ]	[ ]	[ ]
	Did the study report on male female comparison of outcomes?	[ ]	[ ]	[ ]

DECISION OF REVIEWER

	Yes	No	Unsure
1. Is this study potentially relevant for this review?	[ ]	[ ]	[ ]

CONSENSUS

Second review
[ ] Include
[ ] Exclude
[ ] Disagree

Final consensus
[ ] Include
[ ] Exclude
[ ] Disagree
