# Supplementary material for: Understanding the role of sex on outcomes for the cancer patient undergoing treatment with immune checkpoint inhibitors: a scoping review protocol
Source: BMJ Open. 2022 Jul 21;12(7):e059782. doi: 10.1136/bmjopen-2021-059782 (PMC9310149; doi:10.1136/bmjopen-2021-059782)
Supplement: Supplementary data [file bmjopen-2021-059782supp002.pdf]

Extraction/Charting Form

Author(s)*	Study type	Population	ICI 1	ICI 2	Mono/ Combo	Objective/ Outcomes	Summary

* Include year, title, etc.
