# Supplementary material for: Understanding the role of sex on outcomes for the cancer patient undergoing treatment with immune checkpoint inhibitors: a scoping review protocol
Source: BMJ Open. 2022 Jul 21;12(7):e059782. doi: 10.1136/bmjopen-2021-059782 (PMC9310149; doi:10.1136/bmjopen-2021-059782)
Supplement: Reviewer comments [file bmjopen-2021-059782.reviewer_comments.pdf]

PEER REVIEW HISTORY

BMJ Open publishes all reviews undertaken for accepted manuscripts. Reviewers are asked to complete a checklist review form (**http://bmjopen.bmj.com/site/about/resources/checklist.pdf**) and are provided with free text boxes to elaborate on their assessment. These free text comments are reproduced below.

ARTICLE DETAILS

**TITLE (PROVISIONAL)**	Understanding the Role of Sex on Outcomes for the Cancer Patient Undergoing Treatment with Immune Checkpoint Inhibitors: A Scoping Review Protocol
**AUTHORS**	Shaver, Amy; Nikita, Nikita; Sharma, Swapnil; Lefler, Daniel; Basu-Mallick, Atrayee; Johnson, Jennifer; Butryn, ML; Lu-Yao, Grace

VERSION 1 – REVIEW

**REVIEWER**	Mandi Pratt-Chapman<br>George Washington University, GW Cancer Center
**REVIEW RETURNED**	30-Jan-2022

**GENERAL COMMENTS**	Study design:<br>1. What are the implications of your review on transgender and intersex individuals with neither female-exclusive nor male-exclusive hormones?<br>2. Why are patients not invited to comment on study design or relevant outcomes?<br><br>Abstract<br>1. Typo: First instance of "effects" should be "affects"; second instance should remain "effects."<br>2. Page 5/ Stage 4: Missing a word in line 20

**REVIEWER**	Xue-Song Liu<br>ShanghaiTech University
**REVIEW RETURNED**	20-Mar-2022

**GENERAL COMMENTS**	Shaver et al. plan to perform a review of sex on the outcomes for the cancer patient undergoing immunotherapy with ICI. Overall this is an interesting topic for scientific community. One Key issue of this study is that the search terms and the mentioned datasets are not sufficient, and many studies could be missed under the proposed search strategy.<br><br>1, The names of ICI drugs, for example nivolumab, BMS-936558, pembrolizumab, MK-3475, atezolizumab, MPDL3280A, durvalumab, MEDI4736, avelumab, MSB0010718C, BMS-936559, cemiplimab, and REGN2810……. should be included in the search terms;<br><br>2, the included databases are not sufficient, and many abstracts should also be included, for example, abstracts in the American Society of Clinical Oncology (ASCO), the European Society for Medical Oncology (ESMO), and the American Association for Cancer Research (AACR).

	3, “sex differences” should also be included as search terms.<br><br>4, a spelling mistake in line 31, effects-> affects

**REVIEWER**	Amanda Friend<br>University of Leeds
**REVIEW RETURNED**	07-May-2022

**GENERAL COMMENTS**	This is a useful proposed review on an interesting and currently under-researched area. I have just a few suggestions to aid clarity:<br><br>- Are there any hypotheses as to why women may respond differently<br><br>- Will the initial screen only be carried out by a single researcher, or will two (or more) researchers decide on which papers proceed to full text review?<br><br>- I wonder whether the authors could clarify whether they plan to include papers which do not primarily explore the effects of sex, but which include sex as a co-variate?<br><br>- It seems reasonable to not involve patients/public at this stage, but it may perhaps be useful to reference (if possible) something stating that sex differences in cancer outcomes is known to be important to patients - this could then justify the decision not to involve them further at this stage.<br><br>- I note that the assessment form includes the question "did the study include patients aged 18 or over?" but that this is not mentioned in the inclusion criteria. For clarity, it would be worth including this in the methods section. Will studies including patients both under and over 18 be included, or will only studies solely including patients aged 18 and over be included? This should be clarified (although I accept the number of under 18s on these medications would be minimal).

VERSION 1 – AUTHOR RESPONSE

Reviewer: 1

Dr. Mandi Pratt-Chapman, George Washington University

Comments to the Author:

Study design:

1. What are the implications of your review on transgender and intersex individuals with neither female-exclusive nor male-exclusive hormones?

- *This may highlight the need for more studies that include these individuals explicitly. As a scoping review, we will only be able to include data on existing studies. However, no person has neither female-exclusive nor male-exclusive hormones and there may very well be a predominant hormone at play in both transgender and intersex individuals where exogenous hormones are a key part of treatment. This research may inform as to whether one form of ICI is considered or not, and at the very least it could lead to more informed studies into the area so the patient could make the best-informed decision.*

2. Why are patients not invited to comment on study design or relevant outcomes?

- *We did not believe as we were looking for knowledge gaps in pre-existing literature to include a patient panel. However, future study design involving patients does involve our patient outreach team. We have included the following statement in the 'Patient and public involvement' section': "Any future studies deriving from this work will include patient involvement to help to ensure that, from a patient's perspective, the outcomes are relevant."*

Abstract

1. Typo: First instance of "effects" should be "affects"; second instance should remain "effects."

- *Thank you this has been corrected.*

2. Page 5/ Stage 4: Missing a word in line 20

- *The word 'on' was removed and we believe this corrected the issue.*

Reviewer: 2

Dr. Xue-Song Liu, ShanghaiTech University

Comments to the Author:

Shaver et al. plan to perform a review of sex on the outcomes for the cancer patient undergoing immunotherapy with ICI. Overall this is an interesting topic for scientific community. One Key issue of this study is that the search terms and the mentioned datasets are not sufficient, and many studies could be missed under the proposed search strategy.

1, The names of ICI drugs, for example nivolumab, BMS-936558, pembrolizumab, MK-3475, atezolizumab, MPDL3280A, durvalumab, MEDI4736, avelumab, MSB0010718C, BMS-936559, cemiplimab, and REGN2810……. should be included in the search terms;

- *Thank you for your insight. We will add these key words to the drug classes in the search.*

2, the included databases are not sufficient, and many abstracts should also be included, for example, abstracts in the American Society of Clinical Oncology (ASCO), the European Society for Medical Oncology (ESMO), and the American Association for Cancer Research (AACR).

- *You are correct that abstracts may have been lost; though our search did in fact uncover quite a few abstracts. However, ASCO, ESMO, and AACR publications are also found in PubMed. Our team determined that abstracts do not show the whole picture and often just highlight the "best of" the results that authors want to show; many times leaving out results that could be pertinent to our study. As such, the decision was made to only use full-text publications.*

3, "sex differences" should also be included as search terms.

- *Thank you! "Sex differences" has been included as a search term; however, it should be noted that it is included under the MeSH Term "Sex characteristics".*

4, a spelling mistake in line 31, effects-> affects

- *Thank you for pointing this out; it has been corrected.*

Reviewer: 3

Dr. Amanda Friend, University of Leeds

Comments to the Author:

This is a useful proposed review on an interesting and currently under-researched area. I have just a few suggestions to aid clarity:

- Are there any hypotheses as to why women may respond differently

- *There are a few suggested reasons for the observed differences, including lower tumor mutational burden as well as lower tumor antigenicity. The following sentences have been included. ICIs depend on antigen presentation occurring. However, women sometimes have a lower tumor mutation burden [9]. Another study discovered that in female antigens are not as frequently presented to the immune system by the major histocompatibility complex [10]. Altogether, this may explain why ICIs become less effectual [11].*

- Will the initial screen only be carried out by a single researcher, or will two (or more) researchers decide on which papers proceed to full text review?

- *At least two researchers will review at each article for inclusion. This has been clarified at line 125 in the 'marked' manuscript, as: "Each article will be considered by at least two researchers for inclusion and any discrepancies will be discussed."*

- I wonder whether the authors could clarify whether they plan to include papers which do not primarily explore the effects of sex, but which include sex as a co-variate?

- *Yes, this is part of the full text screen. As an article may mention an ICI and not mention sex but use sex as a covariate in its model. If the study reports the results of the covariate such that the difference between the sexes can be compared than the study will be included. A sentence of clarification has been added, "An important part of full-text screening is to discern the inclusion of sex comparison, which my only occur through covariate inclusion but still offer comparison between the sexes."*

- It seems reasonable to not involve patients/public at this stage, but it may perhaps be useful to reference (if possible) something stating that sex differences in cancer outcomes is known to be important to patients - this could then justify the decision not to involve them further at this stage.

- *Thank you for this comment. The statement has been added to the Patient and public involvement section.*

- I note that the assessment form includes the question "did the study include patients aged 18 or over?" but that this is not mentioned in the inclusion criteria. For clarity, it would be worth including this in the methods section. Will studies including patients both under and over 18 be included, or will only studies solely including patients aged 18 and over be included? This should be clarified (although I accept the number of under 18s on these medications would be minimal).

- *Only studies including patients age 18 and over will be included. The inclusion criteria now reads, "This scoping review will only include adult patients aged 18 years or older…"*

VERSION 2 – REVIEW

**REVIEWER**	Xue-Song Liu<br>ShanghaiTech University
**REVIEW RETURNED**	18-Jun-2022

**GENERAL COMMENTS**	My previous points have been addressed in the revised manuscript.

**REVIEWER**	Amanda Friend<br>University of Leeds
**REVIEW RETURNED**	11-Jun-2022

**GENERAL COMMENTS**	Many thanks for responding to the suggested comments. I am happy to recommend this paper for publication, and look forward to reading the completed review, which I am sure will make a useful addition to the literature.
