# Supplementary material for: Understanding the role of sex on outcomes for the cancer patient undergoing treatment with immune checkpoint inhibitors: a scoping review protocol
Source: BMJ Open. 2022 Jul 21;12(7):e059782. doi: 10.1136/bmjopen-2021-059782 (PMC9310149; doi:10.1136/bmjopen-2021-059782)
Supplement: Author's manuscript [file bmjopen-2021-059782.draft_revisions.pdf]

BMJ Open is committed to open peer review. As part of this commitment we make the peer review history of every article we publish publicly available.

When an article is published we post the peer reviewers' comments and the authors' responses online. We also post the versions of the paper that were used during peer review. These are the versions that the peer review comments apply to.

The versions of the paper that follow are the versions that were submitted during the peer review process. They are not the versions of record or the final published versions. They should not be cited or distributed as the published version of this manuscript.

BMJ Open is an open access journal and the full, final, typeset and author-corrected version of record of the manuscript is available on our site with no access controls, subscription charges or pay-per-view fees (http://bmjopen.bmj.com).

If you have any questions on BMJ Open's open peer review process please email info.bmjopen@bmj.com

BMJ Open

# Understanding the Role of Sex on Outcomes for the Cancer Patient Undergoing Treatment with Immune Checkpoint Inhibitors: A Scoping Review Protocol


| | |
|---|---|
| Journal: | *BMJ Open* |
| Manuscript ID | bmjopen-2021-059782 |
| Article Type: | Protocol |
| Date Submitted by the Author: | 30-Nov-2021 |



| | |
|---|---|
| Complete List of Authors: | Shaver, Amy; Thomas Jefferson University Sidney Kimmel Medical College, Department of Medical Oncology<br>Nikita, Nikita; Thomas Jefferson University Sidney Kimmel Medical College, Department of Medical Oncology<br>Sharma, Swapnil; Thomas Jefferson University Sidney Kimmel Medical College, Department of Medical Oncology<br>Lefler, Daniel; Thomas Jefferson University Sidney Kimmel Medical College, Department of Medical Oncology<br>Basu-Mallick, Atrayee; Thomas Jefferson University Sidney Kimmel Medical College, Department of Medical Oncology<br>Johnson, Jennifer; Thomas Jefferson University Sidney Kimmel Medical College, Department of Medical Oncology<br>Butryn, ML; Drexel University, Department of Psychology<br>Lu-Yao, Grace; Thomas Jefferson University Sidney Kimmel Medical College, Department of Medical Oncology; Jefferson College of Population Health |



| | |
|---|---|
| Keywords: | ONCOLOGY, Immunology < NATURAL SCIENCE DISCIPLINES, Epidemiology < ONCOLOGY |
| | |


SCHOLARONE™
Manuscripts

# Understanding the Role of Sex on Outcomes for the Cancer Patient Undergoing Treatment with Immune Checkpoint Inhibitors: A Scoping Review Protocol

Amy L. Shaver[1], Nikita Nikita[1], Swapnil Sharma[1], Daniel S. Lefler[1], Atrayee Basu-Mallick[1], Jennifer M. Johnson[1], Meghan Butryn[2], Grace Lu-Yao[1,3,*]

[1] Department of Medical Oncology, Sidney Kimmel Cancer Center at Jefferson, Sidney Kimmel Medical College, Philadelphia, PA, USA; Amy.Shaver@Jefferson.edu (A.L.S.), Swapnil.Sharma@Jefferson.edu (S.S.), Fnu.Nikita@Jefferson.edu (N.N.), Daniel.Lefler@Jefferson.edu (D.S.L.), Atrayee.BasuMallick@Jefferson.edu (A.B.), Jennifer.M.Johnson@Jefferson.edu (J.M.J.), Grace Lu-Yao Grace.LuYao@Jefferson.edu (G.L.).

[2] Department of Psychology, Drexel University, Philadelphia, PA, USA; mlb34@drexel.edu (M.B.).

[3] Jefferson College of Population Health, Philadelphia, PA, USA.

[*] Correspondence: Grace.LuYao@Jefferson.edu.

## Abstract

### Introduction

Immune checkpoint inhibitors (ICIs) have changed the treatment landscape for multiple cancer types. Sex plays an important role in both the development of cancer as well as the functioning of the immune system. Though a difference in response to immune therapy is emerging between men and women it is unclear how this difference effects cancer outcomes and what the potential underlying mechanisms are for those effects.

### Method and analysis

The framework for this methodology was guided by the Joanna Briggs Institute Manual for Evidence Synthesis. Two independent researchers will screen titles and abstracts followed by full-text screening for manuscript inclusion. Full length studies describing the influence of sex differences on cancer outcomes in patients treated with ICIs will be included. After data is extracted it will be summarized for presentation.

### Ethics and dissemination

The findings of this scoping review will be published in a peer-reviewed journal. The results will be used to inform future studies on the potential differential impacts of immune checkpoint inhibitors. All data are from published openly accessible sources and therefore no ethical clearance is necessary. The protocol is also registered on https://figshare.com.

**Keywords:** Immunotherapy, oncology, sex differences

Word count: 1,219

*Strength and limitations of this study*

- Multidisciplinary team utilized during the planning and design
- Multiple databases utilized to source literature
- No formal quantitative synthesis completed without sufficient data

For peer review only

## Introduction

Immune checkpoint inhibitors (ICIs) have demonstrated clinical efficacy in several solid tumor types [1]. Furthermore, the role of ICIs is going to be investigated in the neoadjuvant, adjuvant, and definitive settings in ongoing clinical trials making it not only important to understand how to improve patient's abilities to respond to treatment as it expands its role in multiple cancer types.

Sex effects both the innate and adaptive immune system responses, however less than 10% of all immunology-related studies consider sex while reporting results [2, 3]. Sex hormones have been shown to influence immune system functioning. Among patients with autoimmune diseases medications used to suppress immune response were more efficacious in men than women while medications to stimulate response are more efficacious in women [4]. Sex plays an important role in the pathogenesis and prognosis of several cancer types. For a vast majority of cancer types, men have a higher predisposition of developing cancer than women. Specifically, men have a two-fold higher risk of mortality from all malignant cancer types than women (after excluding sex-specific cancers such as breast and prostate) [5]. Women are underrepresented in RCTs for immunotherapies. In a meta-analysis of phase II and III immunotherapy, RCTs observed improvement in overall survival and progression free survival for both men and women, but the benefit was much larger among men than in women [6-8]. Since evidence suggests that men and women respond differently to therapies, it is important to explore these differences to redefine clinical decision making and improve outcomes for all patients with cancer. A first step of that discovery is through an examination of the current evidence.

The purpose of this scoping review is to describe the influence that sex has on the outcomes experienced by cancer patients on immune checkpoint inhibitor therapy and to identify and analyze any knowledge gaps in the field.

## Methods

### Protocol design

This scoping review will be conducted according to the Joanna Briggs Institute methodology for scoping reviews which is based predominately on the protocols established by Arksey and O'Malley but includes the revisions suggested by Levac et al and Peters et al [9-13]. The review will follow six steps including: 1) defining the research question, 2) identifying relevant studies, 3) study selection, 4) charting the data, 5) collating, summarizing, and reporting the results, and 6) consultation. Reporting of findings will be conducted according to Preferred Reporting Items for Systematic Reviews and Meta-Analyses (PRISMA) guidelines using the PRISMA extension for scoping reviews (PRISMA-ScR) checklist [14]. The protocol is also registered on https://figshare.com.

**Stage 1: Identifying the research question**

The central research question for this review is: what influence does sex play on the outcomes experienced by patients with cancer on immune checkpoint inhibitor therapy? Outcomes are being left purposefully vague so as to capture as much information as possible. All outcome data will then be categorized later in the 'collating, summarizing, and reporting' stage. Also to be examined is whether the immune checkpoint inhibitor therapy is monotherapy, dual therapy or combination with other therapies.

**Stage 2: Identifying relevant studies, Search Strategy**

The search strategy for this review is the result of prior research in the adjacent fields of prostate cancer, lung cancer, and immunotherapy as well as the strategies recommended by Tawfik et al for adapting searches according to database [15]. An experienced search librarian was also consulted. We will conduct a search of PubMed, CINAHL, Cochrane Library, and Scopus.

For peer review only

We will conduct a search using the following keywords: cancer, “neoplasms”, “immune checkpoint inhibitors”, “sex”, “sex factors”, and associated mesh terms. These terms will be combined with the Boolean operators “AND” and “OR”.

The initial search will be in PubMed. A similar search will be used for Cochrane which also utilizes MeSH terms. Only key words will be used for SCOPUS. CINAHL utilizes Subject terms in lieu of MeSH terms.

The search string for the PubMed database is as follows:

cancer* OR ("Neoplasms"[Mesh])

AND

"Sex"[MeSH Terms] OR "Sex Characteristics"[MeSH Terms] OR "Sex Factors"[MeSH Terms] OR "Male"[MeSH Terms] OR "Female"[MeSH Terms] OR “sex-related” OR “gender-related”

AND

“Immune checkpoint inhibitor” OR “anti-PD-1” OR “anti-CTLA-4” OR “anti-PD-L1” OR “Immune checkpoint blockade”

## Stage 3: Study selection

### Inclusion Criteria

*Types of participants*
This scoping review will only include patients being treated with immune checkpoint inhibitors (ICIs) for those cancers for which the Food and Drug Administration (FDA) has approved the use of ICIs as treatment as of December 2020.

*Concept*
The review will focus on peer-reviewed publications that seek to clarify the influence of sex on the outcomes of immune checkpoint inhibitors.

*Context*
The review will include both institutional and community care settings.

*Types of sources*
All peer-reviewed publications published since 2010 through October 2021.

*Exclusion Criteria*
The search will be restricted to articles and reports published in English.

Opinion pieces.

Letters to the editor.

Studies identified by these terms which satisfy the inclusion criteria will be considered for the initial title and abstract screening. The search string will be adapted for other databases as required. The reference lists of all included articles will be searched for additional studies. As required by good practice, the completed strings for each database will be included in the published scoping review.

If further information is required, we will contact authors of the publications as appropriate.

For peer review only

The research team will use Endnote X9 software for managing imported references and removing duplications. The title, abstracts and keywords for all articles will be screened by two independent reviewers from the research team to determine whether they satisfy the inclusion criteria. Uncertainties, will be discussed.

Articles satisfying the initial screen will undergo full text screening by two independent researchers from the research team. Disagreements of study eligibility will be resolved through discussion with a senior member of the research team.

**Stage 4: Charting the data**

## Extraction of the results

Three members of the research team will participate in the data extraction process. From each article the following information will be extracted: author, year of publication, title, ICI, study type/design, study population, primary objective(s), and outcome(s)/summary.

## Patient and public involvement

This research will be done without patient or involvement. Patients are neither invited to comment on the study design nor consulted to develop patient-relevant outcomes nor to interpret nor disseminate the results.

**Stage 5: Collating, Summarizing, and Reporting**

## Presentation of our results

Our search results will be presented in a PRISMA flowchart and an appended PRISMA-Scr checklist. The extracted data will be presented under the following headings: author, year of publication, study type, study population, primary objective(s), and outcome(s).

A full summary of evidence including an overview of concepts and types of evidence available as well as a discussion of limitations and our conclusions will follow. Analyses of mono and combination therapies will be conducted separately when possible. We will identify gaps in the literature and highlight the implications for future research.

## Stage 6: Consultation

We have purposefully included researchers from multiple disciplines in our group (pharmacy, medicine, and epidemiology). The diversity of the group brings fresh views and broad experiences to the analysis of the literature. At the end of the study a final consultation will take place so the results of the study can have the context of clinical practice knowledge.

## Ethics and dissemination

The scoping review as indicated earlier is based upon openly accessible published material and is therefore not subject to an ethical review board. The findings of this scoping review will be published in a peer-reviewed journal. The results will be used to inform future studies on the potential differential impacts of immune checkpoint inhibitors.

## Author contributions

Study concept and design: ALS, NN, SS, DSL, AB, JMJ, MB, GL

Protocol writing and review: ALS, NN, GL

## Funding statement

This work was supported, in part, by the NCI Cancer Center Support Grant 5P30CA056036.

For peer review only

## Competing interests statement

The authors declare that they have no competing interests.

## Data availability statement

All data relevant to the study will be included in the published manuscript of results as text or as supplementary information.

For peer review only

## References


1. Antonia, S.J., et al., *Durvalumab after Chemoradiotherapy in Stage III Non–Small-Cell Lung Cancer.* New England Journal of Medicine, 2017. **377**(20): p. 1919-1929.
2. Klein, S.L. and K.L. Flanagan, *Sex differences in immune responses.* Nat Rev Immunol, 2016. **16**(10): p. 626-38.
3. Beery, A.K. and I. Zucker, *Sex bias in neuroscience and biomedical research.* Neurosci Biobehav Rev, 2011. **35**(3): p. 565-72.
4. Klein, S.L. and R. Morgan, *The impact of sex and gender on immunotherapy outcomes.* Biol Sex Differ, 2020. **11**(1): p. 24.
5. Cook, M.B., et al., *Sex Disparities in Cancer Mortality and Survival.* Cancer Epidemiology Biomarkers & Prevention, 2011. **20**(8): p. 1629-1637.
6. Botticelli, A., et al., *The sexist behaviour of immune checkpoint inhibitors in cancer therapy?* Oncotarget, 2017. **8**(59): p. 99336-99346.
7. Wu, Y., et al., *Correlation between sex and efficacy of immune checkpoint inhibitors (PD-1 and CTLA-4 inhibitors).* Int J Cancer, 2018. **143**(1): p. 45-51.
8. Conforti, F., L. Pala, and A. Goldhirsch, *Different effectiveness of anticancer immunotherapy in men and women relies on sex-dimorphism of the immune system.* Oncotarget, 2018. **9**(58): p. 31167-31168.
9. Peters, M.D., et al., *Chapter 11: Scoping reviews.* Joanna Briggs Institute reviewer's manual, 2021: p. 408-446.
10. Arksey, H. and L. O'Malley, *Scoping studies: towards a methodological framework.* International Journal of Social Research Methodology, 2005. **8**(1): p. 19-32.
11. Levac, D., H. Colquhoun, and K.K. O'Brien, *Scoping studies: advancing the methodology.* Implement Sci, 2010. **5**: p. 69.
12. Peters, M.D., et al., *Guidance for conducting systematic scoping reviews.* Int J Evid Based Healthc, 2015. **13**(3): p. 141-6.
13. Peters, M.D., *In no uncertain terms: the importance of a defined objective in scoping reviews.* JBI Database System Rev Implement Rep, 2016. **14**(2): p. 1-4.
14. Tricco, A.C., et al., *PRISMA Extension for Scoping Reviews (PRISMA-ScR): Checklist and Explanation.* Ann Intern Med, 2018. **169**(7): p. 467-473.
15. Tawfik, G.M., et al., *A step by step guide for conducting a systematic review and meta-analysis with simulation data.* Tropical medicine and health, 2019. **47**: p. 46-46.


For peer review only

**Inclusion Assessment Form**
Sex and immune checkpoint inhibitor (ICI) treatment

Reviewer: ______________________________ Date: ______________

Reference: ______________________________________________

__________________________________________________________

Instructions: Complete the form on each study. If the final decision is "no", exclude that study.

**CRITERIA**

| | Yes | No | Unsure |
|---|---|---|---|
| 1. Study Publication | | | |
| Is the study a full manuscript? | [ ] | [ ] | [ ] |
| Was the study published between January 2010 and November 2021? | [ ] | [ ] | [ ] |
| Was the study published in English? | [ ] | [ ] | [ ] |
| 2. Study Population | | | |
| Did the study include patients aged 18 and over? | [ ] | [ ] | [ ] |
| 3. Study Exposure | | | |
| Were patients receiving treatment with an ICI? | [ ] | [ ] | [ ] |
| Does the study include both males and females? | [ ] | [ ] | [ ] |
| 4. Study Outcomes | | | |
| Did the study report on one or more outcomes?<br>i.e. The study is not an opinion, editorial, etc. only | [ ] | [ ] | [ ] |

**DECISION OF REVIEWER**

| | Yes | No | Unsure |
|---|---|---|---|
| 1. Is this study potentially relevant for this review? | [ ] | [ ] | [ ] |

**CONSENSUS**

Second review
[ ] Include
[ ] Exclude
[ ] Disagree

Final consensus
[ ] Include
[ ] Exclude
[ ] Disagree

For peer review only

**Extraction/Charting Form**

| Author(s)* | Study type | Population | ICI 1 | ICI 2 | Mono/ Combo | Objective/ Outcomes | Summary |
|---|---|---|---|---|---|---|---|
| | | | | | | | |
| | | | | | | | |
| | | | | | | | |

* Include year, title, etc.

For peer review only

## Preferred Reporting Items for Systematic reviews and Meta-Analyses extension for Scoping Reviews (PRISMA-ScR) Checklist

| SECTION | ITEM | PRISMA-ScR CHECKLIST ITEM | REPORTED ON PAGE # |
|---|---|---|---|
| **TITLE** | | | |
| Title | 1 | Identify the report as a scoping review. | 1 |
| **ABSTRACT** | | | |
| Structured summary | 2 | Provide a structured summary that includes (as applicable): background, objectives, eligibility criteria, sources of evidence, charting methods, results, and conclusions that relate to the review questions and objectives. | 1 |
| **INTRODUCTION** | | | |
| Rationale | 3 | Describe the rationale for the review in the context of what is already known. Explain why the review questions/objectives lend themselves to a scoping review approach. | 2 |
| Objectives | 4 | Provide an explicit statement of the questions and objectives being addressed with reference to their key elements (e.g., population or participants, concepts, and context) or other relevant key elements used to conceptualize the review questions and/or objectives. | 2 |
| **METHODS** | | | |
| Protocol and registration | 5 | Indicate whether a review protocol exists; state if and where it can be accessed (e.g., a Web address); and if available, provide registration information, including the registration number. | 1-4 |
| Eligibility criteria | 6 | Specify characteristics of the sources of evidence used as eligibility criteria (e.g., years considered, language, and publication status), and provide a rationale. | 3 |
| Information sources* | 7 | Describe all information sources in the search (e.g., databases with dates of coverage and contact with authors to identify additional sources), as well as the date the most recent search was executed. | 2 |
| Search | 8 | Present the full electronic search strategy for at least 1 database, including any limits used, such that it could be repeated. | 3 |
| Selection of sources of evidence† | 9 | State the process for selecting sources of evidence (i.e., screening and eligibility) included in the scoping review. | 3-4 |
| Data charting process‡ | 10 | Describe the methods of charting data from the included sources of evidence (e.g., calibrated forms or forms that have been tested by the team before their use, and whether data charting was done independently or in duplicate) and any processes for obtaining and confirming data from investigators. | 4 |
| Data items | 11 | List and define all variables for which data were sought and any assumptions and simplifications made. | 4 |
| Critical appraisal of individual sources of evidence§ | 12 | If done, provide a rationale for conducting a critical appraisal of included sources of evidence; describe the methods used and how this information was used in any data synthesis (if appropriate). | 4 |

For peer review only

| SECTION | ITEM | PRISMA-ScR CHECKLIST ITEM | REPORTED ON PAGE # |
|---|---|---|---|
| Synthesis of results | 13 | Describe the methods of handling and summarizing the data that were charted. | 4 |
| **RESULTS – INCOMPLETE UNTIL STUDY COMPLETED** | | | |
| Selection of sources of evidence | 14 | Give numbers of sources of evidence screened, assessed for eligibility, and included in the review, with reasons for exclusions at each stage, ideally using a flow diagram. | N/A |
| Characteristics of sources of evidence | 15 | For each source of evidence, present characteristics for which data were charted and provide the citations. | N/A |
| Critical appraisal within sources of evidence | 16 | If done, present data on critical appraisal of included sources of evidence (see item 12). | N/A |
| Results of individual sources of evidence | 17 | For each included source of evidence, present the relevant data that were charted that relate to the review questions and objectives. | N/A |
| Synthesis of results | 18 | Summarize and/or present the charting results as they relate to the review questions and objectives. | N/A |
| **DISCUSSION – INCOMPLETE UNTIL STUDY COMPLETED** | | | |
| Summary of evidence | 19 | Summarize the main results (including an overview of concepts, themes, and types of evidence available), link to the review questions and objectives, and consider the relevance to key groups. | N/A |
| Limitations | 20 | Discuss the limitations of the scoping review process. | 1 |
| Conclusions | 21 | Provide a general interpretation of the results with respect to the review questions and objectives, as well as potential implications and/or next steps. | N/A |
| **FUNDING** | | | |
| Funding | 22 | Describe sources of funding for the included sources of evidence, as well as sources of funding for the scoping review. Describe the role of the funders of the scoping review. | 4 |

JBI = Joanna Briggs Institute; PRISMA-ScR = Preferred Reporting Items for Systematic reviews and Meta-Analyses extension for Scoping Reviews.
* Where *sources of evidence* (see second footnote) are compiled from, such as bibliographic databases, social media platforms, and Web sites.
† A more inclusive/heterogeneous term used to account for the different types of evidence or data sources (e.g., quantitative and/or qualitative research, expert opinion, and policy documents) that may be eligible in a scoping review as opposed to only studies. This is not to be confused with *information sources* (see first footnote).
‡ The frameworks by Arksey and O'Malley (6) and Levac and colleagues (7) and the JBI guidance (4, 5) refer to the process of data extraction in a scoping review as data charting.
§ The process of systematically examining research evidence to assess its validity, results, and relevance before using it to inform a decision. This term is used for items 12 and 19 instead of "risk of bias" (which is more applicable to systematic reviews of interventions) to include and acknowledge the various sources of evidence that may be used in a scoping review (e.g., quantitative and/or qualitative research, expert opinion, and policy document).

*From:* Tricco AC, Lillie E, Zarin W, O'Brien KK, Colquhoun H, Levac D, et al. PRISMA Extension for Scoping Reviews (PRISMAScR): Checklist and Explanation. Ann Intern Med. 2018;169:467–473. doi: 10.7326/M18-0850.

For peer review only

BMJ Open

# Understanding the Role of Sex on Outcomes for the Cancer Patient Undergoing Treatment with Immune Checkpoint Inhibitors: A Scoping Review Protocol


| | |
|---|---|
| Journal: | *BMJ Open* |
| Manuscript ID | bmjopen-2021-059782.R1 |
| Article Type: | Protocol |
| Date Submitted by the Author: | 08-Jun-2022 |
| Complete List of Authors: | Shaver, Amy; Thomas Jefferson University Sidney Kimmel Medical College, Department of Medical Oncology<br>Nikita, Nikita; Thomas Jefferson University Sidney Kimmel Medical College, Department of Medical Oncology<br>Sharma, Swapnil; Thomas Jefferson University Sidney Kimmel Medical College, Department of Medical Oncology<br>Lefler, Daniel; Thomas Jefferson University Sidney Kimmel Medical College, Department of Medical Oncology<br>Basu-Mallick, Atrayee; Thomas Jefferson University Sidney Kimmel Medical College, Department of Medical Oncology<br>Johnson, Jennifer; Thomas Jefferson University Sidney Kimmel Medical College, Department of Medical Oncology<br>Butryn, ML; Drexel University, Department of Psychology<br>Lu-Yao, Grace; Thomas Jefferson University Sidney Kimmel Medical College, Department of Medical Oncology; Jefferson College of Population Health |
| <b>Primary Subject Heading</b>: | Oncology |
| Secondary Subject Heading: | Immunology (including allergy) |
| Keywords: | ONCOLOGY, Immunology < NATURAL SCIENCE DISCIPLINES, Epidemiology < ONCOLOGY |
| | |


SCHOLARONE™
Manuscripts

# Understanding the Role of Sex on Outcomes for the Cancer Patient Undergoing Treatment with Immune Checkpoint Inhibitors: A Scoping Review Protocol

Amy L. Shaver[1], Nikita Nikita[1], Swapnil Sharma[1], Daniel S. Lefler[1], Atrayee Basu-Mallick[1], Jennifer M. Johnson[1], Meghan Butryn[2], Grace Lu-Yao[1,3,*]

[1] Department of Medical Oncology, Sidney Kimmel Cancer Center at Jefferson, Sidney Kimmel Medical College, Philadelphia, PA, USA; Amy.Shaver@Jefferson.edu (A.L.S.), Swapnil.Sharma@Jefferson.edu (S.S.), Fnu.Nikita@Jefferson.edu (N.N.), Daniel.Lefler@Jefferson.edu (D.S.L.), Atrayee.BasuMallick@Jefferson.edu (A.B.), Jennifer.M.Johnson@Jefferson.edu (J.M.J.), Grace Lu-Yao Grace.LuYao@Jefferson.edu (G.L.Y.).

[2] Department of Psychology, Drexel University, Philadelphia, PA, USA; mlb34@drexel.edu (M.B.).

[3] Jefferson College of Population Health, Philadelphia, PA, USA.

[*] Correspondence: Grace.LuYao@Jefferson.edu.

## Abstract

### Introduction

Immune checkpoint inhibitors (ICIs) have changed the treatment landscape for multiple cancer types. Sex plays an important role in both the development of cancer as well as the functioning of the immune system. Though a difference in response to immune therapy is emerging between men and women it is unclear how this difference affects cancer outcomes and what the potential underlying mechanisms are for those effects. The objective of this study is to describe the influence that sex has on the outcomes experienced by cancer patients on immune checkpoint inhibitor therapy and to identify and analyze any knowledge gaps in the field

### Method and analysis

The framework for this methodology was guided by the Joanna Briggs Institute Manual for Evidence Synthesis. The search and review will be conducted from January 2022 through June 2022. Two independent researchers will screen titles and abstracts followed by full-text screening for manuscript inclusion. Full length studies published between 2010 and December 2021 found in PubMed, Cochrane, CINAHL, and Scopus describing the influence of sex differences on cancer outcomes in patients treated with ICIs will be included. After data is extracted it will be summarized for presentation.

### Ethics and dissemination

The findings of this scoping review will be published in a peer-reviewed journal. The results will be used to inform future studies on the potential differential impacts of immune checkpoint inhibitors. All data are from published openly accessible sources and therefore no ethical clearance is necessary. The protocol is also registered on https://figshare.com.

**Keywords:** Immunotherapy, oncology, sex differences

Word count: 2,269

*Strength and limitations of this study*

- Multidisciplinary team utilized during the planning and design

For peer review only

- Multiple databases utilized to source literature
- No formal quantitative synthesis completed without sufficient data

## Introduction

Immune checkpoint inhibitors (ICIs) have demonstrated clinical efficacy in several solid tumor types [1]. Furthermore, the role of ICIs is going to be investigated in the neoadjuvant, adjuvant, and definitive settings in ongoing clinical trials making it not only important to understand how to improve patient's abilities to respond to treatment as it expands its role in multiple cancer types.

Sex effects both the innate and adaptive immune system responses, however less than 10% of all immunology-related studies consider sex while reporting results [2, 3]. Sex hormones have been shown to influence immune system functioning. Among patients with autoimmune diseases medications used to suppress immune response were more efficacious in men than women while medications to stimulate response are more efficacious in women [4]. Sex plays an important role in the pathogenesis and prognosis of several cancer types. For a vast majority of cancer types, men have a higher predisposition of developing cancer than women. Specifically, men have a two-fold higher risk of mortality from all malignant cancer types than women (after excluding sex-specific cancers such as breast and prostate) [5]. Women are underrepresented in RCTs for immunotherapies. In a meta-analysis of phase II and III immunotherapy, RCTs observed improvement in overall survival and progression free survival for both men and women, but the benefit was much larger among men than in women [6-8]. ICIs depend on antigen presentation occurring. However, women sometimes have a lower tumor mutation burden [9]. Another study discovered that in female antigens are not as frequently presented to the immune system by the major histocompatibility complex [10]. Altogether, this may explain why ICIs become less effectual [11]. Since evidence suggests that men and women respond differently to therapies, it is important to explore these differences to redefine clinical decision making and improve outcomes for all patients with cancer. A first step of that discovery is through an examination of the current evidence.

The purpose of this scoping review is to describe the influence that sex has on the outcomes experienced by cancer patients on immune checkpoint inhibitor therapy and to identify and analyze any knowledge gaps in the field.

## Methods

### Protocol design

This scoping review will be conducted according to the Joanna Briggs Institute methodology for scoping reviews which is based predominately on the protocols established by Arksey and O'Malley but includes the revisions suggested by Levac et al and Peters et al [12-16]. The review will follow six steps including: 1) defining the research question, 2) identifying relevant studies, 3) study selection, 4) charting the data, 5) collating, summarizing, and reporting the results, and 6) consultation. Reporting of findings will be conducted according to Preferred Reporting Items for Systematic Reviews and Meta-Analyses (PRISMA) guidelines using the PRISMA extension for scoping reviews (PRISMA-ScR) checklist [17]. The protocol is also registered on https://figshare.com.

**Stage 1: Identifying the research question**

The central research question for this review is: what influence does sex play on the outcomes experienced by patients with cancer on immune checkpoint inhibitor therapy? Outcomes are being left purposefully vague so as to capture as much information as possible. All outcome data will then be categorized later in the 'collating, summarizing, and reporting' stage. Also to be examined is whether the immune checkpoint inhibitor therapy is monotherapy, dual therapy or combination with other therapies.

For peer review only - http://bmjopen.bmj.com/site/about/guidelines.xhtml

**Stage 2: Identifying relevant studies, Search Strategy**

The search strategy for this review is the result of prior research in the adjacent fields of prostate cancer, lung cancer, and immunotherapy as well as the strategies recommended by Tawfik et al for adapting searches according to database [18]. An experienced search librarian was also consulted. We will conduct a search of PubMed, CINAHL, Cochrane Library, and Scopus.

We will conduct a search using the following keywords: cancer, “neoplasms”, “immune checkpoint inhibitors”, “sex”, “sex factors”, and associated mesh terms. These terms will be combined with the Boolean operators “AND” and “OR”.

The initial search will be in PubMed. A similar search will be used for Cochrane which also utilizes MeSH terms. Only key words will be used for SCOPUS. CINAHL utilizes Subject terms in lieu of MeSH terms.

The search string for the PubMed database is as follows:

cancer* OR ("Neoplasms"[Mesh])

AND

"Sex"[MeSH Terms] OR "Sex Characteristics"[MeSH Terms] OR "Sex Factors"[MeSH Terms] OR "Male"[MeSH Terms] OR "Female"[MeSH Terms] OR “sex-related” OR “gender-related” OR “sex differences”

AND

“Immune checkpoint inhibitor” OR “anti-PD-1” OR “anti-CTLA-4” OR “anti-PD-L1” OR “Immune checkpoint blockade” OR “REGN2810” OR “ Cemiplimab” OR “BMS-936559” OR “MSB0010718C” OR “avelumab” OR “MEDI4736” OR “durvalumab” OR MPDL3280A” OR “atezolizumab” OR “MK-3475” OR “pembrolizumab” OR “BMS-936558” OR “nivolumab”

**Stage 3: Study selection**

**Inclusion Criteria**

*Types of participants*

This scoping review will only include adult patients aged 18 years or olderbeing treated with immune checkpoint inhibitors (ICIs) for those cancers for which the Food and Drug Administration (FDA) has approved the use of ICIs as treatment as of December 2020.

*Concept*

The review will focus on peer-reviewed publications that seek to clarify the influence of sex on the outcomes of immune checkpoint inhibitors.

*Context*

The review will include both institutional and community care settings.

*Types of sources*

All peer-reviewed publications published since 2010 through December 2021.

*Exclusion Criteria*

The search will be restricted to articles and reports published in English.

Opinion pieces.

For peer review only

Letters to the editor.

Studies identified by these terms which satisfy the inclusion criteria will be considered for the initial title and abstract screening. The search string will be adapted for other databases as required. The reference lists of all included articles will be searched for additional studies. As required by good practice, the completed strings for each database will be included in the published scoping review.

If further information is required, we will contact authors of the publications as appropriate.

The research team will use Endnote X9 software for managing imported references and removing duplications. The title, abstracts and keywords for all articles will be screened by two independent reviewers from the research team to determine whether they satisfy the inclusion criteria, see Inclusion Assessment Form, supplemental material. Each article will be considered by at least two researchers for inclusion and any discrepancies will be will be discussed.

Articles satisfying the initial screen will undergo full text screening by two independent researchers from the research team. An important part of full-text screening is to discern the inclusion of sex comparison, which my only occur through covariate inclusion but still offer comparison between the sexes. Disagreements of study eligibility will be resolved through discussion with a senior member of the research team.

**Stage 4: Charting the data**

### Extraction of the results

Three members of the research team will participate in the data extraction process. From each article the following information will be extracted: author, year of publication, title, ICI, study type/design, study population, primary objective(s), and outcome(s)/summary, see Extraction Charting Form, supplemental material.

### Patient and public involvement

This research will be done without patient involvement. Patients are neither invited to comment on the study design nor consulted to develop patient-relevant outcomes nor to interpret nor disseminate the results. Sex differences in cancer outcomes are known to be important to patients. Any future studies deriving from this work will include patient involvement to help to ensure that, from a patient's perspective, the outcomes are relevant.

**Stage 5: Collating, Summarizing, and Reporting**

### Presentation of our results

Our search results will be presented in a PRISMA flowchart and an appended PRISMA-Scr checklist. The extracted data will be presented under the following headings: author, year of publication, study type, study population, primary objective(s), and outcome(s).

A full summary of evidence including an overview of concepts and types of evidence available as well as a discussion of limitations and our conclusions will follow. Analyses of mono and combination therapies will be conducted separately when possible. We will identify gaps in the literature and highlight the implications for future research.

### Stage 6: Consultation

We have purposefully included researchers from multiple disciplines in our group (pharmacy, medicine, and epidemiology). The diversity of the group brings fresh views and broad experiences to the analysis of the literature. At the end of the study a final consultation will take place so the results of the study can have the context of clinical practice knowledge.

For peer review only

## Ethics and dissemination

The scoping review as indicated earlier is based upon openly accessible published material and is therefore not subject to an ethical review board. The findings of this scoping review will be published in a peer-reviewed journal. The results will be used to inform future studies on the potential differential impacts of immune checkpoint inhibitors.

## Author contributions

Study concept and design: ALS, NN, SS, DSL, AB, JMJ, MB, GLY

Protocol writing and review: ALS, NN, GLY

## Funding statement

This work was supported, in part, by the NCI Cancer Center Support Grant 5P30CA056036.

## Competing interests statement

The authors declare that they have no competing interests.

## Data availability statement

All data relevant to the study will be included in the published manuscript of results as text or as supplementary information.

For peer review only

## References


1. Antonia, S.J., et al., *Durvalumab after Chemoradiotherapy in Stage III Non–Small-Cell Lung Cancer.* New England Journal of Medicine, 2017. **377**(20): p. 1919-1929.
2. Klein, S.L. and K.L. Flanagan, *Sex differences in immune responses.* Nat Rev Immunol, 2016. **16**(10): p. 626-38.
3. Beery, A.K. and I. Zucker, *Sex bias in neuroscience and biomedical research.* Neurosci Biobehav Rev, 2011. **35**(3): p. 565-72.
4. Klein, S.L. and R. Morgan, *The impact of sex and gender on immunotherapy outcomes.* Biol Sex Differ, 2020. **11**(1): p. 24.
5. Cook, M.B., et al., *Sex Disparities in Cancer Mortality and Survival.* Cancer Epidemiology Biomarkers & Prevention, 2011. **20**(8): p. 1629-1637.
6. Botticelli, A., et al., *The sexist behaviour of immune checkpoint inhibitors in cancer therapy?* Oncotarget, 2017. **8**(59): p. 99336-99346.
7. Wu, Y., et al., *Correlation between sex and efficacy of immune checkpoint inhibitors (PD-1 and CTLA-4 inhibitors).* Int J Cancer, 2018. **143**(1): p. 45-51.
8. Conforti, F., L. Pala, and A. Goldhirsch, *Different effectiveness of anticancer immunotherapy in men and women relies on sex-dimorphism of the immune system.* Oncotarget, 2018. **9**(58): p. 31167-31168.
9. Ye, Y., et al., *Sex-associated molecular differences for cancer immunotherapy.* Nat Commun, 2020. **11**(1): p. 1779.
10. Castro, A., et al., *Strength of immune selection in tumors varies with sex and age.* Nat Commun, 2020. **11**(1): p. 4128.
11. Jang, S.R., et al., *Association Between Sex and Immune Checkpoint Inhibitor Outcomes for Patients With Melanoma.* JAMA Network Open, 2021. **4**(12): p. e2136823-e2136823.
12. Peters, M.D., et al., *Chapter 11: Scoping reviews.* Joanna Briggs Institute reviewer's manual, 2021: p. 408-446.
13. Arksey, H. and L. O'Malley, *Scoping studies: towards a methodological framework.* International Journal of Social Research Methodology, 2005. **8**(1): p. 19-32.
14. Levac, D., H. Colquhoun, and K.K. O'Brien, *Scoping studies: advancing the methodology.* Implement Sci, 2010. **5**: p. 69.
15. Peters, M.D., et al., *Guidance for conducting systematic scoping reviews.* Int J Evid Based Healthc, 2015. **13**(3): p. 141-6.
16. Peters, M.D., *In no uncertain terms: the importance of a defined objective in scoping reviews.* JBI Database System Rev Implement Rep, 2016. **14**(2): p. 1-4.
17. Tricco, A.C., et al., *PRISMA Extension for Scoping Reviews (PRISMA-ScR): Checklist and Explanation.* Ann Intern Med, 2018. **169**(7): p. 467-473.
18. Tawfik, G.M., et al., *A step by step guide for conducting a systematic review and meta-analysis with simulation data.* Tropical medicine and health, 2019. **47**: p. 46-46.


For peer review only

**Inclusion Assessment Form**

Sex and immune checkpoint inhibitor (ICI) treatment

Reviewer: ______________________ Date: ______________

Reference: ______________________________________

## CRITERIA

| | | Yes | No | Unsure |
|---|---|---|---|---|
| 1. Study Publication | | | | |
| | Is the study a full manuscript? | [ ] | [ ] | [ ] |
| | Was the study published between January 2010 and December 2021? | [ ] | [ ] | [ ] |
| | Was the study published in English? | [ ] | [ ] | [ ] |
| 2. Study Population | | | | |
| | Did the study include patients aged 18 and over? | [ ] | [ ] | [ ] |
| 3. Study Exposure | | | | |
| | Were patients receiving treatment with an ICI? | [ ] | [ ] | [ ] |
| | Does the study include both males and females? | [ ] | [ ] | [ ] |
| 4. Study Outcomes | | | | |
| | Did the study report on one or more outcomes? i.e. The study is not an opinion, editorial, etc. only | [ ] | [ ] | [ ] |
| | Did the study report on male female comparison of outcomes? | [ ] | [ ] | [ ] |

## DECISION OF REVIEWER

| | Yes | No | Unsure |
|---|---|---|---|
| 1. Is this study potentially relevant for this review? | [ ] | [ ] | [ ] |

## CONSENSUS

Second review
[ ] Include
[ ] Exclude
[ ] Disagree

Final consensus
[ ] Include
[ ] Exclude
[ ] Disagree

For peer review only

**Extraction/Charting Form**

| Author(s)* | Study type | Population | ICI 1 | ICI 2 | Mono/ Combo | Objective/ Outcomes | Summary |
|---|---|---|---|---|---|---|---|
| | | | | | | | |
| | | | | | | | |
| | | | | | | | |

* Include year, title, etc.

For peer review only

**Preferred Reporting Items for Systematic reviews and Meta-Analyses extension for Scoping Reviews (PRISMA-ScR) Checklist**

| SECTION | ITEM | PRISMA-ScR CHECKLIST ITEM | REPORTED ON PAGE # |
|---|---|---|---|
| **TITLE** | | | |
| Title | 1 | Identify the report as a scoping review. | 1 |
| **ABSTRACT** | | | |
| Structured summary | 2 | Provide a structured summary that includes (as applicable): background, objectives, eligibility criteria, sources of evidence, charting methods, results, and conclusions that relate to the review questions and objectives. | 1 |
| **INTRODUCTION** | | | |
| Rationale | 3 | Describe the rationale for the review in the context of what is already known. Explain why the review questions/objectives lend themselves to a scoping review approach. | 2 |
| Objectives | 4 | Provide an explicit statement of the questions and objectives being addressed with reference to their key elements (e.g., population or participants, concepts, and context) or other relevant key elements used to conceptualize the review questions and/or objectives. | 2 |
| **METHODS** | | | |
| Protocol and registration | 5 | Indicate whether a review protocol exists; state if and where it can be accessed (e.g., a Web address); and if available, provide registration information, including the registration number. | 1-4 |
| Eligibility criteria | 6 | Specify characteristics of the sources of evidence used as eligibility criteria (e.g., years considered, language, and publication status), and provide a rationale. | 3 |
| Information sources* | 7 | Describe all information sources in the search (e.g., databases with dates of coverage and contact with authors to identify additional sources), as well as the date the most recent search was executed. | 2 |
| Search | 8 | Present the full electronic search strategy for at least 1 database, including any limits used, such that it could be repeated. | 3 |
| Selection of sources of evidence† | 9 | State the process for selecting sources of evidence (i.e., screening and eligibility) included in the scoping review. | 3-4 |
| Data charting process‡ | 10 | Describe the methods of charting data from the included sources of evidence (e.g., calibrated forms or forms that have been tested by the team before their use, and whether data charting was done independently or in duplicate) and any processes for obtaining and confirming data from investigators. | 4 |
| Data items | 11 | List and define all variables for which data were sought and any assumptions and simplifications made. | 4 |
| Critical appraisal of individual sources of evidence§ | 12 | If done, provide a rationale for conducting a critical appraisal of included sources of evidence; describe the methods used and how this information was used in any data synthesis (if appropriate). | 4 |

For peer review only

| SECTION | ITEM | PRISMA-ScR CHECKLIST ITEM | REPORTED ON PAGE # |
|---|---|---|---|
| Synthesis of results | 13 | Describe the methods of handling and summarizing the data that were charted. | 4 |
| **RESULTS – INCOMPLETE UNTIL STUDY COMPLETED** | | | |
| Selection of sources of evidence | 14 | Give numbers of sources of evidence screened, assessed for eligibility, and included in the review, with reasons for exclusions at each stage, ideally using a flow diagram. | N/A |
| Characteristics of sources of evidence | 15 | For each source of evidence, present characteristics for which data were charted and provide the citations. | N/A |
| Critical appraisal within sources of evidence | 16 | If done, present data on critical appraisal of included sources of evidence (see item 12). | N/A |
| Results of individual sources of evidence | 17 | For each included source of evidence, present the relevant data that were charted that relate to the review questions and objectives. | N/A |
| Synthesis of results | 18 | Summarize and/or present the charting results as they relate to the review questions and objectives. | N/A |
| **DISCUSSION – INCOMPLETE UNTIL STUDY COMPLETED** | | | |
| Summary of evidence | 19 | Summarize the main results (including an overview of concepts, themes, and types of evidence available), link to the review questions and objectives, and consider the relevance to key groups. | N/A |
| Limitations | 20 | Discuss the limitations of the scoping review process. | 1 |
| Conclusions | 21 | Provide a general interpretation of the results with respect to the review questions and objectives, as well as potential implications and/or next steps. | N/A |
| **FUNDING** | | | |
| Funding | 22 | Describe sources of funding for the included sources of evidence, as well as sources of funding for the scoping review. Describe the role of the funders of the scoping review. | 4 |

JBI = Joanna Briggs Institute; PRISMA-ScR = Preferred Reporting Items for Systematic reviews and Meta-Analyses extension for Scoping Reviews.

* Where *sources of evidence* (see second footnote) are compiled from, such as bibliographic databases, social media platforms, and Web sites.

† A more inclusive/heterogeneous term used to account for the different types of evidence or data sources (e.g., quantitative and/or qualitative research, expert opinion, and policy documents) that may be eligible in a scoping review as opposed to only studies. This is not to be confused with *information sources* (see first footnote).

‡ The frameworks by Arksey and O'Malley (6) and Levac and colleagues (7) and the JBI guidance (4, 5) refer to the process of data extraction in a scoping review as data charting.

§ The process of systematically examining research evidence to assess its validity, results, and relevance before using it to inform a decision. This term is used for items 12 and 19 instead of "risk of bias" (which is more applicable to systematic reviews of interventions) to include and acknowledge the various sources of evidence that may be used in a scoping review (e.g., quantitative and/or qualitative research, expert opinion, and policy document).

*From:* Tricco AC, Lillie E, Zarin W, O'Brien KK, Colquhoun H, Levac D, et al. PRISMA Extension for Scoping Reviews (PRISMAScR): Checklist and Explanation. Ann Intern Med. 2018;169:467–473. doi: 10.7326/M18-0850.

For peer review only
